# Scalable Fabrication of Perovskite Solar Cells with Inkjet-Printed Perovskite Absorbers Processed under Ambient Conditions

**DOI:** 10.1021/acsami.4c20567

**Published:** 2025-04-29

**Authors:** Dongli Lu, Mahboubeh Jamshidi, James M. Gardner, Liubov Belova

**Affiliations:** † Department of Materials Science and Engineering, KTH Royal Institute of Technology, Stockholm 10044, Sweden; ‡ Department of Chemistry, Applied Physical Chemistry, KTH Royal Institute of Technology, Stockholm 10044, Sweden

**Keywords:** inkjet printing, perovskite solar cells, in situ heat treatment, solvent engineering, ink additive

## Abstract

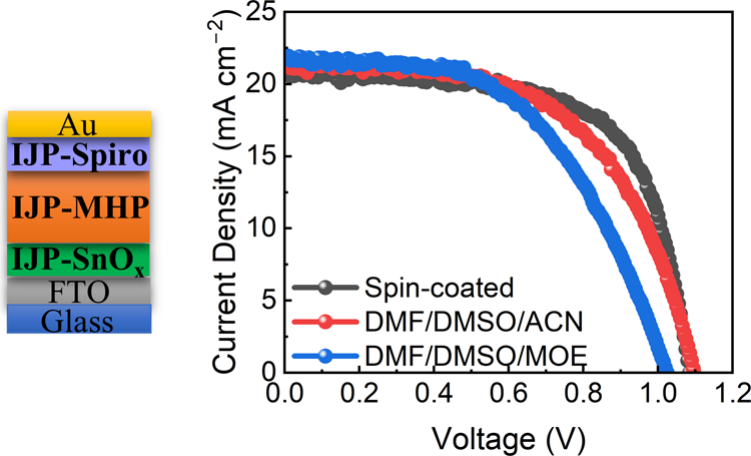

It is still a challenge to fabricate a smooth, high-quality perovskite film under ambient conditions via an inkjet printing process. Here, we report a systematic study of the fabrication of one-step inkjet-printed metal halide perovskite (IJP-MHP) films under ambient conditions. An inkjet-printed perovskite film with full surface coverage and large columnar grains was obtained through in situ heat treatment, self-vapor annealing (self-VA), and solvent engineering. An efficiency of 13.44% was achieved for the perovskite solar cell (PSC) with an architecture of FTO/IJP-SnO_*x*_/IJP-MHP/IJP-spiro-OMeTAD/Au. Furthermore, an ink additive polyvinylpyrrolidone (PVP) and antisolvent extraction treatment were carried out to retard perovskite nucleation and grain growth. Resulting perovskite films with improved film morphology and uniformity were obtained, although they delivered no competitive performance. Further optimization can be conducted to improve efficiency by careful selection of ink additives.

## 1 Introduction

Metal halide perovskites (MHPs) have emerged as promising light absorbers for photovoltaic applications because of their high absorption coefficient, long diffusion length, low exciton binding energy, and fast carrier mobility.^1−5^ In the past decade, the power conversion efficiency (PCE) of perovskite solar cells (PSCs) has increased remarkably from 3.8 to 26.7%.^6,7^ However, MHPs are usually fabricated by spin-coating under controlled inert atmospheres (e.g., a glovebox filled with nitrogen), which is not beneficial for the upscaling and commercialization of PSCs. Therefore, a low-cost and scalable fabrication method for PSCs is highly required.

Inkjet printing is a promising technique for large-scale manufacturing with negligible material wastage. Its drop-on-demand (DOD) technology allows for high material utilization, which is particularly important in reducing toxic Pb-contained waste. Furthermore, DOD inkjet printing can achieve precise digital control over ink droplets, enabling direct patterning, which has great potential for the additive manufacturing of multijunction solar cells and integrated circuits. Additionally, its noncontact and maskless properties make the fabrication process simple and straightforward. Inkjet-printed MHP (IJP-MHP) absorbers were first reported by Wei and co-workers, who prepared a combined ink of carbon and methylammonium iodide (MAI, MA = CH_3_NH_3_^+^) to simultaneously deposit the carbon electrode and drop MAI on PbI_2_ to form in situ MAPbI_3_ using an inkjet printing technique.^8^ A PCE of 11.60% was delivered by the inkjet-printed carbon-based planar PSCs with a configuration of FTO/TiO_2_/MAPbI_3_/C. Later, Li et al. successfully fabricated one-step inkjet-printed MAPbI_3_ films with the aid of in situ heat treatment and MACl additive and obtained a PCE of 12.3% for the cell FTO/c-TiO_2_/m-TiO_2_/MAPbI_3_/spiro-OMeTAD/Au.^9^ Recently, Eggers et al. achieved an optimum PCE of 21.6% and a stabilized PCE of 18.5% for p-i-n PSCs (ITO/NiO_*x*_/perovskite/C_60_/BCP/Au) with inkjet-printed triple-cation mixed halide perovskite (Cs_0.10_FA_0.75_MA_0.15_Pb(Br_0.15_I_0.85_)_3_, FA = CH(NH_2_)_2_^+^) layers, which exhibited large columnar grains and yielded a thickness larger than 1 μm.^10^

To further promote the upscaling manufacturing of PSCs, there is a need for investigations on the inkjet printing of all functional layers within PSCs, i.e., perovskite absorbers, charge transport layers, and electrodes. Gheno et al. reported inkjet-printed PSCs fully processed under ambient conditions at temperatures lower than 100 °C and achieved a PCE of 10.7% for the device with the structure of ITO/IJP-WO_*x*_/IJP-perovskite/IJP-spiro-OMeTAD/Au.^11^ Verma et al. fabricated mesoscopic carbon-based PSCs with the configuration of FTO/IJP-c-TiO_2_/IJP-m-TiO_2_/IJP-m-ZrO_2_/C/IJP-perovskite under ambient conditions and obtained large-area PSCs (1.5 cm^2^) with a PCE of 9.1%, measured for an active area of 0.64 cm^2^.^12^ Chalkias et al. also demonstrated inkjet-printed carbon-based PSCs based on air-processed inkjet-printed perovskite layers, wherein the “coffee-ring” effect was suppressed.^13^ As a result, submodules with a 34.2 cm^2^ active area with an architecture of FTO/IJP-c-TiO_2_/IJP-m-TiO_2_/IJP-perovskite/C exhibited a mean PCE of 9.09%, along with outstanding stability and low upscaling losses. In addition, an inverted p-i-n-architecture PSC with all-IJP triple-cation perovskite and charge transport layers was reported.^14^ The inverted PSC with the structure of ITO/IJP-NiO_*x*_/IJP-perovskite/IJP-PCBM/IJP-BCP/Au offered a high PCE above 17% as well as excellent operational stability (40 h) at elevated temperature (85 °C). The reports presented above are focused on the fabrication of PSCs with all-IJP active layers, excluding electrodes (bottom or/and top electrode). Recently, Gao et al. achieved fully inkjet-printed inverted PSCs with the cell structure of IJP-Ag/IJP-PEDOT:PSS/IJP-perovskite/IJP-PC_71_BM/IJP-PEI/IJP-Ag on flexible PEN substrates.^15^ Moreover, their large-area devices, with an effective area of 120 cm^2^, exhibited a high PCE of 16.78% with excellent stability when stored in air without packaging. There are no further reports on fully inkjet-printed PSCs as the fabrication of all functional layers via inkjet printing is uniquely challenging.

In the conventional spin-coating approach, an antisolvent is dripped onto the in situ perovskite film during spin-coating, leading to the formation of a transparent thin film with an AX-PbI_2_-DMSO (A = MA^+^ or FA^+^, X = I^+^, Br^+^, or Cl^+^) intermediate phase.^16,17^ Upon annealing, this intermediate phase film turns out to be a black, smooth perovskite film. Thus, the aim of the solvent-quenching process is to induce fast solvent extraction and facilitate the formation of a compact and smooth intermediate phase film.^16,18^ In the inkjet printing process, without centrifugal force, it is difficult to uniformly drip an antisolvent onto the as-inkjet-printed perovskite film.^19^ As a result, inkjet-printed perovskite films usually undergo slow solvent evaporation process and uncontrolled nucleation and crystallization processes as excess solvent cannot be quickly removed. This results in the formation of rough perovskite films with large grains and even incomplete surface coverage. Therefore, the main challenge for the fabrication of inkjet-printed perovskite films is to achieve high-quality perovskite films with proper crystallization and flat morphology. Various attempts made to control the nucleation and crystallization dynamics of inkjet-printed perovskite films can be categorized as ink engineering,^20−22^ inkjet processing engineering (including optimizing printing parameters and substrate conditions),^23,24^ and post-treatment engineering.^19^ The ink formulation including ink composition, solvent system, and additives determines the rheological and solvent evaporation characteristics of the perovskite precursor ink, thereby influencing the surface morphology and crystallization of the resulting perovskite film.^25−28^ In situ heat treatment or thermal annealing post-treatment is usually utilized to speed up solvent evaporation and boost perovskite crystallization.^9,19^ Printing parameters such as drop spacing and printing passes can also control the thickness, grain size, and surface roughness of the resulting perovskite film.^29−31^ Most of the attempts described above involve the use of vacuum-assisted post-treatment or a clean room (controlled inert) atmosphere for the fabrication of inkjet-printed perovskite films, which can increase the complexity and cost of large-scale production of PSCs. Thus, more attention should be paid to inkjet-printed perovskite layers processed in air. Furthermore, there is a lack of systematic study of how these optimization strategies influence the formation and crystallization dynamics of inkjet-printed perovskite films processed under ambient conditions. To the best of our knowledge, studies on SnO_2_-based PSCs with inkjet-printed perovskite absorbers and inkjet-printed charge transport layers processed under ambient conditions are also absent.

In this work, we developed a scalable and reproducible fabrication method for mixed-cation metal halide perovskite films FA_0.85_MA_0.15_PbI_2.55_Br_0.45_ processed under ambient conditions via an inkjet printing process. To obtain a uniform, fully covered perovskite film with proper crystallization, the crystallization dynamics of inkjet-printed perovskite films were systematically investigated by manipulating the ink formulation, substrate (printing) temperature, and post-treatment. Nucleation of the jetted perovskite solution was enhanced through in situ heat treatment, which played a significant role in the formation of a fully covered perovskite film. With increasing substrate temperature, the resulting perovskite films exhibited improved surface coverage and increased grain size, with full surface coverage achieved at 150 °C. Crystallization was further promoted by a post-treatment—self-vapor annealing—which facilitated crystal growth and increased grain size. This effect was attributed to nonequilibrium formation and decomposition of DMSO-containing intermediate phases at grain boundaries under a DMSO vapor atmosphere. The nucleation and crystallization processes were also influenced by the ink formulation of the perovskite precursor solution. Through solvent engineering, a ternary solvent system of DMF/DMSO/ACN yielded optimal results. The addition of ACN not only improved the wetting properties of the jetted solution but also facilitated rapid solvent evaporation, promoting substantial nucleation and enhancing crystal growth.

In combination with inkjet-printed charge transport layers, a PCE of 13.44% was delivered by the cell with the architecture of FTO/IJP-SnO_*x*_/IJP-MHP/IJP-spiro-OMeTAD/Au. This efficiency was comparable and even higher than the reported values for n-i-p PSCs with inkjet-printed functional layers.^11−13^ In addition, uniform and smooth perovskite films were obtained using the ink additive PVP and antisolvent extraction treatment, although they produced low PCEs. The device performance of these uniform inkjet-printed perovskite films can be improved by further optimization through properly selecting ink additives.

## 2 Results and Discussion

**Figure 1:**
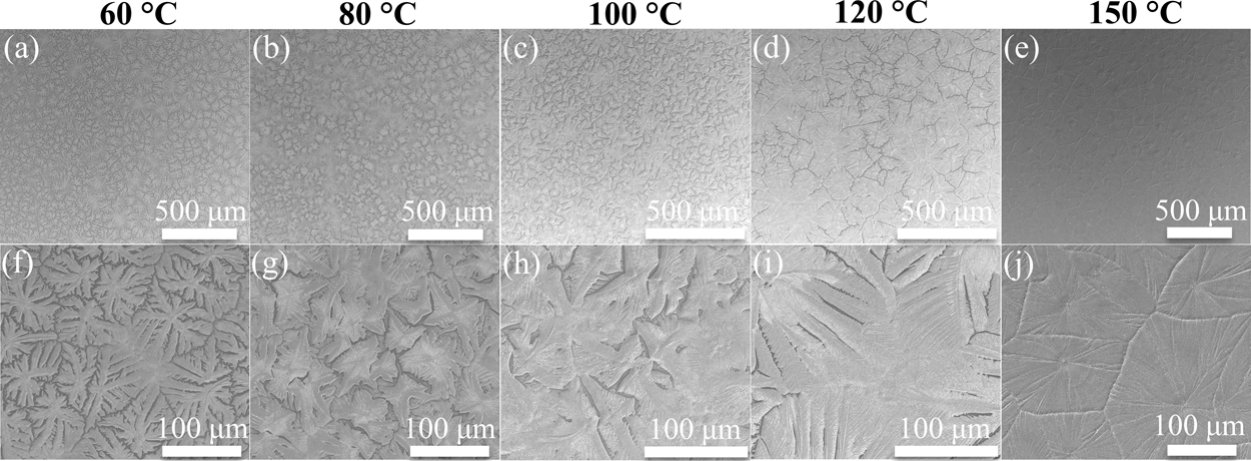
(a–e) SEM images of perovskite films printed at various substrate temperatures. The expanded images are shown in (f–j).

Due to the absence of convective spreading flow and antisolvent extraction during the inkjet printing process, we used in situ heat treatment instead to speed up the solvent evaporation and induce perovskite nucleation. This in situ heat treatment was achieved by jetting droplets of perovskite precursors to a heated substrate, which was placed on a stage with a temperature controller (Figure S1). The optimum in situ heat treatment condition was related to the solvent system used for perovskite deposition. Here, the commonly used solvent composition of dimethylformamide (DMF) and dimethyl sulfoxide (DMSO) was used initially. The substrates were heated to different temperatures, and after printing, these substrates were immediately transferred to a hot plate. Then, all of the as-prepared perovskite films were annealed at a commonly used temperature of 100 °C for 30 min. The morphology of the resulting perovskite films was examined using a scanning electron microscope (SEM). As shown in Figure 1, the perovskite film exhibited a surface morphology with leaf-like islands and yielded poor coverage over the underlying substrate, when the substrate temperature was 60 °C. This phenomenon was attributed to the slow solvent evaporation process during inkjet printing due to the high boiling points of DMF and DMSO (153 °C for DMF and 189 °C for DMSO). The slow supersaturation of the jetted precursor solution led to a slow nucleation process and the formation of a small number of island-shaped nuclei.^32^ The following crystal growth of these nuclei resulted in the formation of large island-shaped perovskite grains. Unlike the spin-coating process, the formation and morphology of the resulting films via the inkjet printing process were not only determined by the volatility of the solvent system but also influenced by the rheological properties, such as viscosity and surface tension. Specifically, the jetted droplets were not pinned after dwelling on the substrate due to the low substrate temperature. These unpinned droplets were subject to shape shrinkage induced by the high viscosity and surface tension of the precursor solution, resulting in island features of the resulting perovskite films. With the substrate temperature increasing from 60 to 150 °C, the perovskite film exhibited increased crystal size and improved surface coverage. At a substrate temperature of 150 °C, the perovskite film achieved full coverage over the substrate and showed a large crystal size of hundreds of micrometers. A nucleation center was observed in the middle of each crystal grain. Therefore, in situ heat treatment boosted supersaturation of the jetted solution and enabled the formation of a substantial number of nuclei, which was the key to produce a perovskite film that fully covered the substrate.^33^

**Figure 2:**
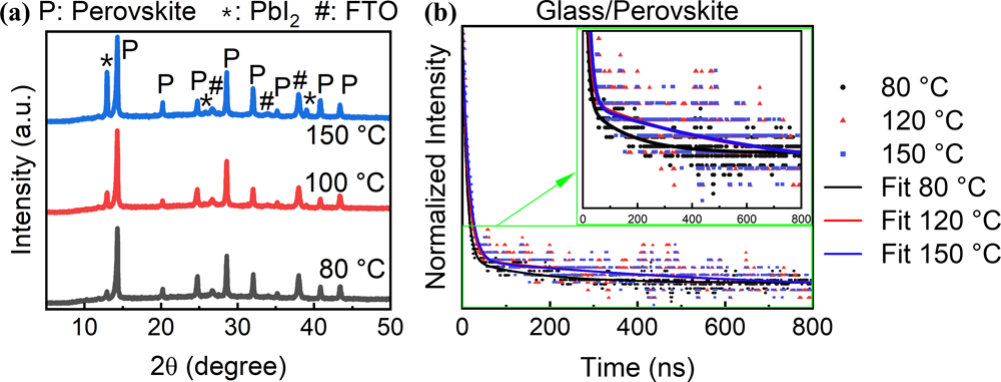
(a) XRD patterns and (b) TRPL with corresponding fitting kinetic traces of perovskite films printed at different substrate temperatures.

Figure 2a demonstrates X-ray diffraction (XRD) patterns of the perovskite films printed at different substrate temperatures. The intensity of the XRD characteristic peak at 14.2° increased slightly with the substrate temperature increasing from 80 to 150 °C. This indicated that there is no significant difference in the crystallinity of the perovskite films. However, the ratio of PbI_2_ (12.9°) to perovskite increased dramatically at a substrate temperature of 150 °C, which represented the presence of a large amount of excess PbI_2_. Since the annealing conditions were fixed for all the perovskite films printed at different substrate temperatures, the increased amount of excess PbI_2_ rarely came from the decomposition of perovskite during the annealing process. It was more like it formed during inkjet printing. The high substrate temperature enabled fast intermolecular exchange between organic halide molecules and DMSO coordinated in the intermediate phase, causing the sublimation of organic halide molecules to form PbI_2_.^17^ This excess PbI_2_ could passivate perovskite grain boundaries and interfaces and benefit efficiencies.^34,35^ The XRD results also showed that proper crystallization of the perovskite films happened when annealed at 100 °C as no other impurity phases were detected. When the annealing temperature was further increased to 140 °C, severe decomposition occurred, and a vast quantity of PbI_2_ phase was detected (Figure S2). We adopted this commonly used annealing temperature (100 °C) for processing perovskite films in this work.

The film quality of the inkjet-printed perovskite films was further examined by time-resolved photoluminescence (TRPL) measurements. The TRPL decay curves are shown in Figure 2b, which were fitted with a biexponential decay function, as described in the experimental part. The fitted results are summarized in Table 1. The longer slow decay of τ_2_ represented a slower recombination rate of photogenerated carriers in the inkjet-printed perovskite film. The average lifetime increased with the substrate temperature increasing from 80 to 150 °C, suggesting improved film quality with less defects acting as recombination centers. This was consistent with the increased crystal grain size and improved surface coverage as aforementioned.

**Table 1:** TRPL Decay Lifetime of Inkjet-Printed Perovskite Films with In Situ Heat Treatment at Different Substrate Temperatures

	substrate temperature (°C)	*A*_1_	τ_1_ (ns)	*A*_2_	τ_2_ (ns)	τ_ave_ (ns)
glass/IJP-perovskite	80	0.907 ± 0.012	9.9 ± 0.2	0.093 ± 0.004	207.0 ± 23.9	27.6
	120	0.896 ± 0.020	12.7 ± 0.5	0.104 ± 0.007	314.2 ± 87.6	42.8
	150	0.900 ± 0.016	14.8 ± 0.5	0.100 ± 0.010	421.9 ± 141.5	55.5

**Figure 3:**
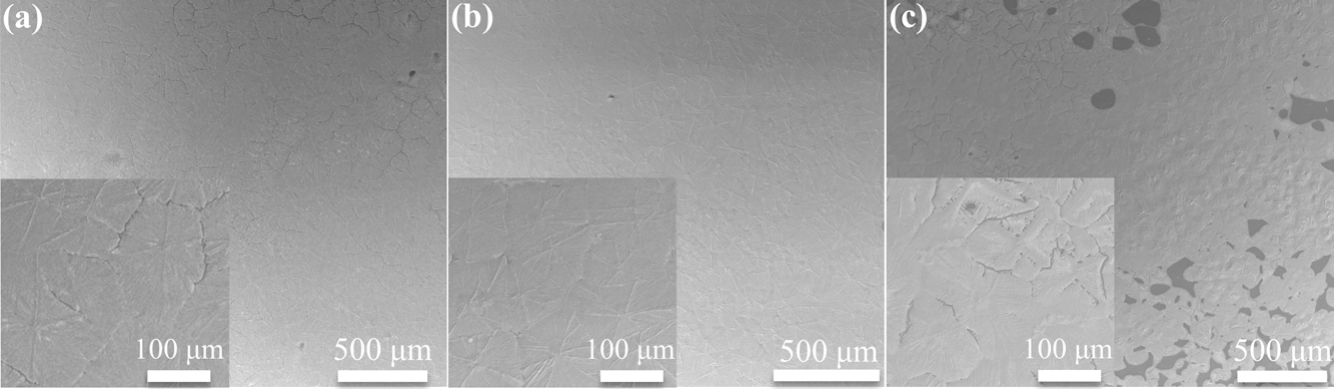
SEM images of perovskite films (a) without, (b) with self-VA, and (c) with VA treatment. These perovskite films were printed at a substrate temperature of 140 °C.

Postannealing treatment of the as-prepared perovskite films can also influence crystal growth. Apart from vacuum-assisted post-treatment, vapor-assisted thermal annealing (VA) has been reported as an effective approach to boost crystal growth and increase grain size.^36−38^ We examined two different vapor-assisted treatments, VA and self-VA treatment. The corresponding procedures are described in the experimental section. For comparison, a reference sample was thermally annealed for only 30 min without any VA treatment. As seen in Figure 3a, the thermally annealed film followed a normal growth process as discussed above and did not offer full coverage over the substrate at a substrate temperature of 140 °C. For self-VA treatment, solvent vapor gradually evaporated from the perovskite film and filled in the Petri dish. The solvent vapor molecule can dissolve the perovskite film and remelt grain boundaries, which bonded adjacent grains.^37^ The origin of this phenomenon could be attributed to the nonequilibrium formation and decomposition of the DMSO-coordinated intermediate phase at perovskite grain boundaries under a DMSO atmosphere, thereby facilitating grain boundary migration and enabling the formation of large crystal grains.^39^ The perovskite film was densified and almost exhibited full coverage, as shown in Figure 3b. The DMSO-annealed perovskite films showed large pinholes, which were unevenly distributed on the substrate (Figure 3c). This was due to the high solvent vapor concentration condition and nonuniform distribution of the added solvent vapor inside the Petri dish. It is a challenge to precisely add the appropriate amount of solvent and realize the uniform distribution of the solvent when applying VA treatment. For self-VA treatment, the solvent vapor was almost distributed evenly. Therefore, self-VA treatment can retain the advantages of solvent annealing and benefit the reproducibility of the printed perovskite films.

**Figure 4:**
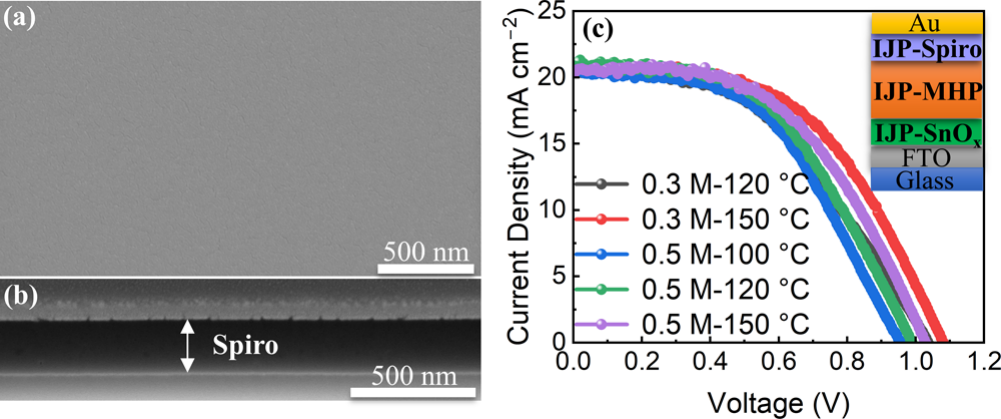
(a,b) Top-view and cross-sectional FIB/SEM images of the inkjet-printed spiro-OMeTAD film. (c) Reverse-scan *J*–*V* characteristics of photovoltaic parameters of the cells with perovskite films printed at different substrate temperatures.

The effects of substrate temperature on the surface morphology, perovskite crystallization, and film quality implied that the in situ heat treatment was of great importance to the growth and formation of inkjet-printed perovskite films. Then, PSCs with printed perovskite absorbers were fabricated to investigate the effect of the substrate temperature on device performance. A SnO_*x*_ electron transport layer (ETL) was fabricated as reported in our previous work.^40^ Regarding the fabrication of hole transport layers (HTLs), the detailed procedure is described in the experimental section. We used toluene instead of chlorobenzene as the solvent for spiro-OMeTAD to improve printability and accelerate evaporation of spiro-OMeTAD inks. A compact, smooth inkjet-printed HTL was obtained, as shown in Figure 4a,b. The top electrode Au was thermally evaporated as usual. A schematic of the resulting PSCs with inkjet-printed MHPs and charge transport layers is shown in Figure 4c. The effects of substrate temperature on the *J*–*V* characteristics and the distribution of photovoltaic parameters are illustrated in Figures 4c and S3. For inkjet-printed perovskite films prepared from 0.5 M inks, when the substrate temperature was increased from 100 to 150 °C, the PSC device exhibited a PCE increasing from 9.73% (8.92% ± 0.68%) to 10.94% (9.75% ± 0.88%) (Table S1). The improvement mainly originated from the increase in the open-circuit voltage (*V*_OC_) and short-circuit current density (*J*_SC_), which were partially attributed to improved coverage and suppressed recombination due to avoiding direct contact between the charge transport layers. The increases in *V*_OC_ and *J*_SC_ were also associated with variations in the thickness of the perovskite film. As shown in Figure S4a–c, the thickness of perovskite grains decreased by 50% when the substrate temperature was increased from 100 to 150 °C. This was owing to improved grain nucleation and growth and suppressed shape shrinkage as aforementioned. The literature suggests that perovskite films have an optimal thickness of 500 nm,^41^ and thicker films can increase the internal series resistance and hinder charge transport. In response, we used perovskite precursor inks with a lower concentration of 0.3 M to reduce film thickness. The perovskite films printed from 0.3 M inks exhibited a dramatic decrease in thickness (Figure S4d,e). Consequently, an improved optimum PCE of 11.71% was achieved for the device with a perovskite film printed from a 0.3 M ink and printed at 150 °C, compared with that of the 0.5 M-150 °C sample (10.94%). However, the average efficiency (9.60% ± 1.39%) decreased slightly, and a broad PCE distribution was observed (Figure S3), which was due to the presence of some quite thin areas. The 0.3 M–150 °C sample exhibited a higher PCE than the 0.3 M–120 °C sample, which also verified that the device performance improved with the substrate temperature. In addition, the grain size of all the inkjet-printed perovskite films was larger than the thickness of the films, which would be beneficial for charge transport since the charges could transport through perovskite grains without encountering grain boundaries along the vertical orientation. However, there existed a large variety of film thicknesses from the centers to the edges of the perovskite grains (Figure S4). At some areas, the perovskite grains with very large thickness can increase internal resistance, leading to charge recombination. That was why the fill factor (FF) was relatively low (<0.55).

**Figure 5:**
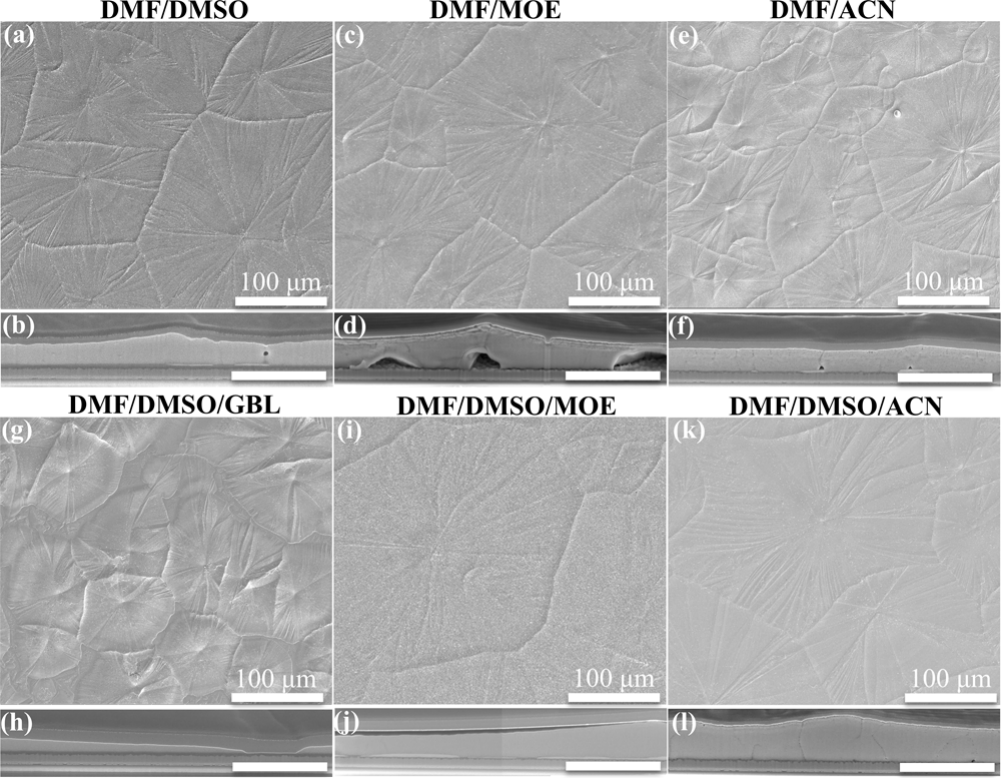
(a,c,e,g,i,k) Top-view and (b,d,f,h,j,l) cross-sectional SEM images of perovskite films printed with precursor inks consisting of different solvent systems. The scale bars in the cross-sectional images are 3 μm.

To further improve perovskite film quality, we adjusted the ink formulation by using solvents with low boiling points or/and low viscosity and surface tension. 2-Methoxyethanol (MOE) and acetonitrile (ACN) have been reported as solvents for uniform perovskite deposition.^32,42,43^ Herein, we replaced DMSO with MOE or ACN to make a mixed solvent of DMF/MOE or DMF/ACN with the same volume ratio of 4/1 as the commonly used combination of DMF/DMSO. Figure 5 shows the top-view and cross-sectional SEM images of the perovskite films prepared by using inks with different solvent systems. MOE has a lower boiling point (124 °C) with similar viscosity and surface tension compared with DMSO. The perovskite film prepared from DMF/MOE exhibited similar morphology to that prepared from DMF/DMSO, whereas large caves were observed at the interface between the perovskite film with the underlying SnO_*x*_ film (Figure 5c,d). This is a wetting problem that can be mitigated by introducing solvents with low viscosity and low surface tension, which indicates that the mixed solvent DMF/MOE with only lower boiling point was not beneficial for the formation of a functional inkjet-printed perovskite layer for PSCs.

**Figure 6:**
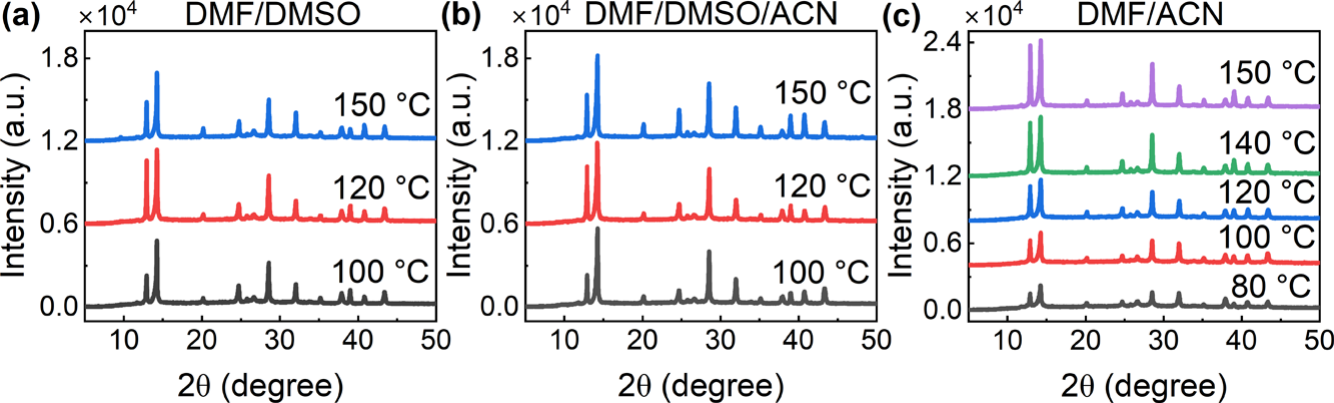
(a–c) XRD patterns of perovskite films printed with precursor inks consisting of different solvent compositions: DMF/DMSO, DMF/DMSO/ACN, and DMF/ACN.

ACN possesses a much lower viscosity and surface tension as well as a lower boiling point (82 °C) in comparison with DMSO. Thus, the perovskite film prepared from DMF/ACN exhibited a uniform thickness, although smaller crystal grains were observed.^43^ As seen in XRD patterns (Figure 6c), the peak ratio of PbI_2_ to perovskite was high, even at relatively low substrate temperatures for the perovskite films prepared from the DMF/ACN solvent system. This phenomenon could be assigned to the absence of DMSO and was also observed in the XRD patterns of the DMF/MOE perovskite films (Figure S5). This further verified the importance of DMSO, which can form a strong coordination with PbI_2_ to produce an intermediate phase, thereby facilitating the conversion of PbI_2_ to the perovskite phase and preventing the excessive amount of unreacted PbI_2_.^16,44−46^ Besides, PbI_2_ is insoluble in ACN, which might also result in unreacted PbI_2_ residual in the resultant films.^42^ Consequently, we kept DMSO and just added MOE or ACN as the third solvent into the solvent system. The two perovskite films shown in Figure 5i,k exhibited larger grains than the perovskite film prepared with the original solvent DMF/DMSO. The more volatile solvent MOE or ACN facilitated faster solvent evaporation, enabled the formation of an adequate number of nuclei, and simultaneously boosted crystal grain growth. The XRD patterns further confirmed that the perovskite films printed from the solvent system DMF/DMSO/ACN exhibited similar crystallization as those from the solvent system DMF/DMSO (Figure 6b and Table S2). However, in the cross-section of the perovskite film, thickness variety still existed, and the smoothness was not significantly improved (Figure 5j,l). In comparison, a high-boiling-point solvent, γ-butyrolactone (GBL, 204 °C), was tested and added to the solvent system DMF/DMSO. The resulting perovskite film poorly covered the substrate (Figure 5g,h).

**Figure 7:**
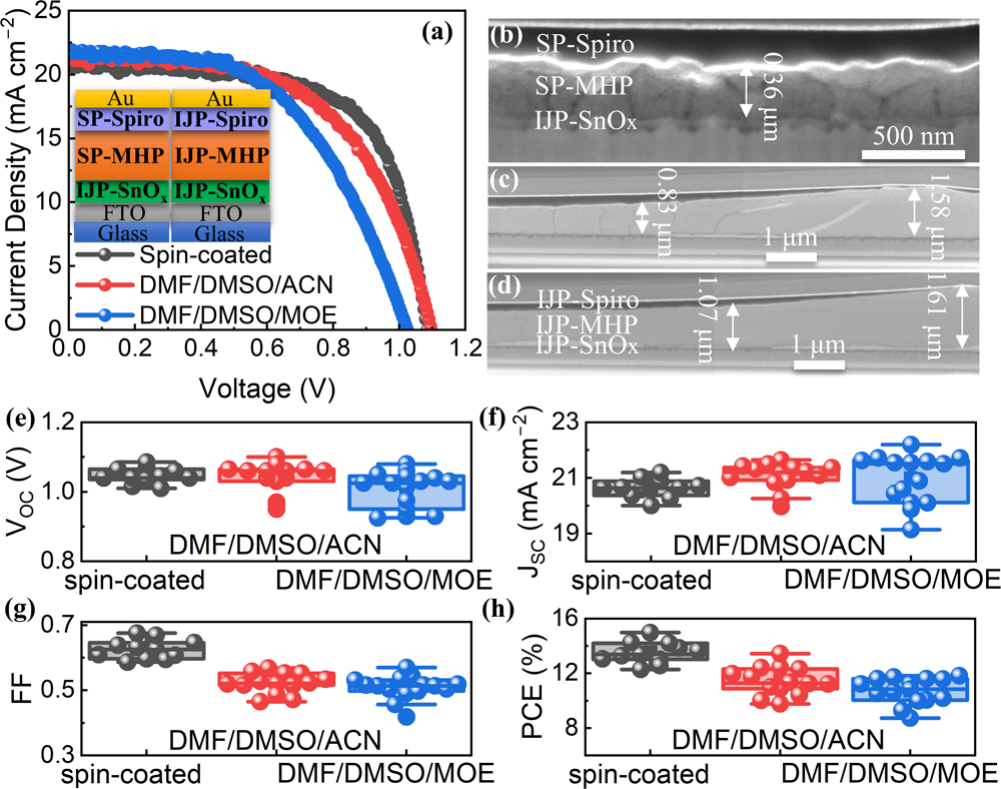
(a) *J*–*V* characteristics, (b–d) cross-sectional SEM images, and (e–h) distributions of photovoltaic parameters of PSCs with spin-coated perovskite layers and with inkjet-printed perovskite layers prepared from two different solvent systems, DMF/DMSO/ACN and DMF/DMSO/MOE.

PSCs were then fabricated using perovskite films prepared from DMF/DMSO/ACN and DMF/DMSO/MOE. The PSCs using spin-coated perovskite films (SP-MHPs) were also tested as a reference. Figure 7a shows the *J*–*V* characteristics of these PSCs. The device with the solvent system of DMF/DMSO/ACN achieved a PCE of 13.44% with a *V*_OC_ of 1.10 V, a *J*_SC_ of 21.50 mA cm^–2^, and an FF of 0.568, while the one with the DMF/DMSO/MOE solvent system offered a lower PCE of 11.82% with a *V*_OC_ of 1.03 V, a *J*_SC_ of 21.72 mA cm^–2^, and an FF of 0.531. In contrast, the spin-coated device delivered the highest PCE of 14.97% with a *V*_OC_ of 1.09 V, a *J*_SC_ of 20.64 mA cm^–2^, and an FF of 0.669. The average PCEs followed the same trend (Table S3 and Figure 7e–h). As seen in Figure S6b, the spin-coated perovskite layer exhibited nanosized crystal grains (200–500 nm), which were much smaller than those with hundreds of micrometers in the inkjet-printed perovskite films. Moreover, a polycrystalline microstructure was observed in the cross-section of the spin-coated perovskite layer, whereas single-crystalline grains were observed through the thickness of the inkjet-printed perovskite layer (Figure 7b,c). Large perovskite grains without boundaries in the vertical orientation can enable fast charge transport. This could be the reason why the inkjet-printed PSC with the solvent DMF/DMSO/ACN achieved higher *V*_OC_ and *J*_SC_ than those of the spin-coated PSC. On the other hand, the spin-coated perovskite layer possessed a smooth surface morphology with an even, homogeneous thickness, whereas the uneven, inkjet-printed perovskite layers caused the uneven deposition of the overlying spiro-OMeTAD and Au layers. As a result, the inkjet-printed devices exhibited FFs much lower than those of the spin-coated devices. Besides, the thicker inkjet-printed perovskite layers could also lead to lower FFs.

**Figure 8:**
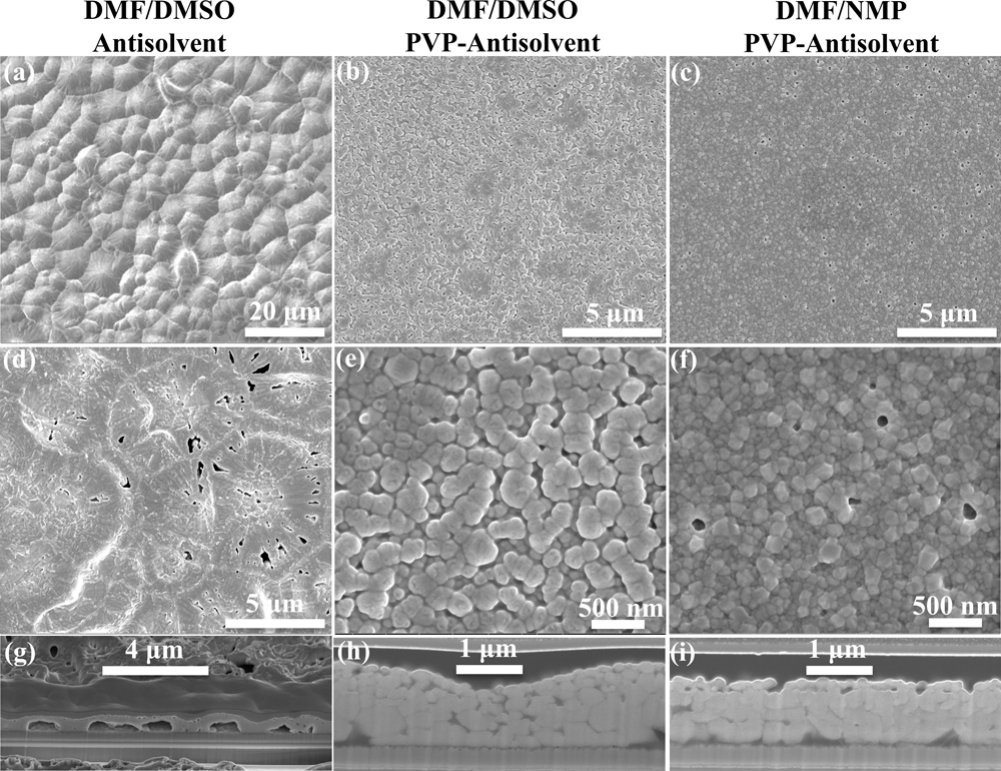
SEM images of inkjet-printed perovskite films with a DMF/DMSO solvent system and antisolvent treatment (a), with a DMF/DMSO-PVP solvent system and antisolvent treatment (b), and with a DMF/NMP-PVP solvent system and antisolvent treatment (c). (d–f) Corresponding expanded SEM images of images (a–c) with large magnification. (g–i) Cross-sectional SEM images of the three corresponding perovskite films.

The use of in situ heat treatment and solvent engineering accelerated solvent evaporation and boosted crystal growth during the inkjet printing process, which enabled the formation of continuous perovskite films with large grains of hundreds of micrometers. On the other hand, the uncontrolled perovskite nucleation and growth resulted in a rough film with large thickness variety. In addition, a large amount of excess PbI_2_ formed at the high substrate temperature could induce ion migration and hysteresis and deteriorate the photostability in the long term.^47^ To realize a uniform perovskite film with improved morphology, we introduced a room-temperature nonvolatile additive polyvinylpyrrolidone (PVP) into the DMF/DMSO solvent system to slow down solvent evaporation and regulate perovskite nucleation and growth during inkjet printing. The weight ratio of the added PVP to PbI_2_ was fixed at 5%. The new perovskite precursor ink was printed at a relatively low substrate temperature of 80 °C to form a wet film. This obtained wet film was then treated with an antisolvent chlorobenzene bath to extract solvents and form a solid intermediate film. After subsequent thermal annealing, a crystallized perovskite film was obtained. Perovskite films with antisolvent treatment and without PVP additives were also prepared as comparison. As seen in Figure 8a,d,g, it can be found that antisolvent treatment enabled the formation of a continuous perovskite film with full surface coverage, even at a relatively low substrate temperature (80 °C), at which the perovskite film without antisolvent treatment exhibited incomplete surface coverage (Figure S7a–c). However, there were many caves at the interface between the perovskite layer and the underlying SnO_*x*_ layer, which can be detrimental to photovoltaic performance. Upon introducing PVP into the precursor inks, the perovskite film exhibited an improved morphology with small grains (100–200 nm), as shown in Figure 8b,e. In the cross-section of this film (Figures 8h and S8a), the large caves disappeared, and the mitigated thickness variety was also observed, indicating improved uniformity. Furthermore, DMSO was replaced by *N*-methyl-2-pyrrolidinone (NMP) that evaporated more slowly than DMSO. Figure 8c,f shows that the grain size was further decreased, and the film uniformity was further improved (Figures 8i and S8b).

The above results can be explained as follows. The antisolvent extraction and annealing processes can induce perovskite growth from the top and bottom sides, respectively. Without PVP, the fast solvent extraction induced the formation and growth of perovskite from the top side, which caused separation at the perovskite/SnO_*x*_ interface.^48^ After PVP was added, the stable time of the yellow wet film increased, and the crystallization of perovskite from the top side was slowed down during the antisolvent bath process. Therefore, the separation was suppressed, leading to good physical contact at the perovskite/SnO_*x*_ interface. The wave-shaped cross-section for the DMF/DMSO-PVP-Anti case was due to existing perovskite growth from the top side. For the DMF/NMP-PVP-Anti case, due to the lower volatility of NMP, perovskite growth was further suppressed during the antisolvent extraction and annealing processes, thereby enabling improved uniformity and smoothness. This implies that the introduction of PVP additives retarded solution supersaturation, perovskite nucleation, and crystal growth during inkjet printing, which allowed for controlling the following antisolvent extraction and enabling the formation of a uniform perovskite film.^49^ It should be noted that the perovskite crystals aligned randomly and that there were considerable grain boundaries in both vertical and horizontal directions upon adding PVP. This was attributed to the coexistence of the downward and upward crystal growth induced by the antisolvent extraction and annealing processes, respectively, resulting in structural mismatch and formation of grain boundaries in all directions.^48^ In comparison, the crystals aligned along the vertical direction, and no obvious grain boundaries were observed in the horizontal direction for the control perovskite film without PVP additives or antisolvent treatment. The unaligned crystal and grain boundaries deteriorate PSC device performance. In addition, there were dark areas between grains in the cross-sections of the perovskite films with PVP additive, which was the residual PVP that could hinder charge transport.

**Figure 9:**
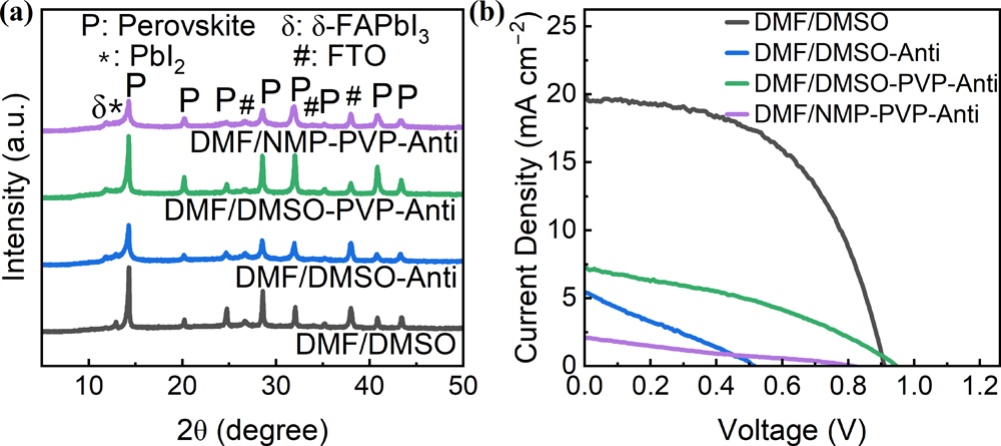
(a) XRD of printed perovskite films with ink additive PVP and antisolvent extraction treatment. (b) *J*–*V* curves of PSCs using inkjet-printed perovskite films with ink additive PVP and antisolvent extraction treatment.

X-ray diffraction measurements were conducted to investigate the crystallization of the inkjet-printed perovskite films with a PVP additive and antisolvent treatment (Figure 9a). Typical characteristic peaks of perovskite were observed in the DMF/DMSO-PVP-Anti case as same as in the DMF/DMSO case without any treatment. The obvious difference was the change of crystal orientation. In the DMF/DMSO case, the strongest peak at 14.3° represented a preferable crystal orientation along [110], owing to the crystal growth from the bottom side upon annealing. In the DMF/DMSO-PVP-Anti case, the intensity of the diffraction peaks at 14.3° and 24.8° decreased slightly while the peaks at 20.2°, 32.0°, and 40.8° exhibited more pronounced intensity. This arose from the crystal growth from both the up and down sides as discussed above. A new weak peak at 11.8° corresponding to the δ-FAPbI_3_ phase was detected, but the peak at 12.9° for PbI_2_ was almost absent in the DMF/DMSO-PVP-Anti case. This suggested that the transformation of PbI_2_ to perovskite was improved, although an unfavorable δ-FAPbI_3_ phase was formed. The intensity of the characteristic peaks decreased significantly for the DMF/NMP-PVP-Anti case, indicating less crystallinity or smaller grains, as observed in the SEM images. This could be assigned to the absence of DMSO. The lack of a DMSO-related intermediate phase was not beneficial for the crystallinity of perovskite. In the case of DMF/DMSO-Anti, the perovskite film exhibited lower XRD intensity, although its XRD spectral features resembled those of the DMF/DMSO-PVP-Anti case, which further confirmed the negative effect of introducing the antisolvent extraction process in the case without the PVP additive.

To further examine the quality of the perovskite films with the PVP additive and antisolvent treatment, TRPL measurements were conducted (Figure S9). The fitting results are listed in Table S4. The DMF/DMSO perovskite film exhibited the shortest fast decay τ_1_ and the longest slow decay τ_2_, implying faster charge transport and less recombination losses in the large-grain film. The DMF/DMSO-PVP-Anti perovskite film also possessed shorter τ_1_ and longer τ_2_ than the DMF/NMP-PVP-Anti perovskite film, which could be due to a larger-grained morphology with fewer pinholes in the surface. The longest fast decay and shortest slow decay were observed for the DMF/DMSO-Anti perovskite film, which was assigned to its poor interfacial contact.

PSCs were then fabricated to examine the photovoltaic performance of the treated perovskite films. Figure 9b shows *J*–*V* curves of these PSCs treated by the PVP additive and an antisolvent bath. Detailed photovoltaic parameters are summarized in Table S5. Unfortunately, the devices exhibited poor performance in either the DMF/DMSO-PVP case or the DMF/NMP-PVP case. Their *V*_OC_ values were comparable with those of the DMF/DMSO case, whereas the *J*_SC_ values were much lower than those of the working devices. This was probably due to the presence of the PVP additive that was electrically insulating. The higher PCEs of the DMF/DMSO-PVP devices than those of the DMF/NMP-PVP devices could be attributed to larger grains, enhanced crystallinity, suppressed recombination, and improved charge transport. Besides, the poor performance of the DMF/DMSO-Anti devices was assigned to the inhomogeneous morphology and poor physical contact between the perovskite layer and the ETL. Overall, compared with the untreated perovskite film with uncontrolled nucleation and crystal growth, the perovskite films with the PVP additive and antisolvent treatment exhibited significantly improved morphology with improved uniformity. This makes these inkjet-printed perovskite films promising for large-scale applications in PSCs. Therefore, further work should be conducted to improve device performance by using electrically conductive additives or additives that can be removed by an antisolvent bath treatment.

## 3 Conclusions

In summary, this study demonstrates a systematic investigation of the scalable fabrication of perovskite films under ambient conditions via a one-step inkjet printing process. Through in situ heat treatment and self-VA treatment, continuous perovskite films with full coverage and large columnar grains were obtained. With further optimization via solvent engineering, a PCE of 13.44% was achieved for PSCs with the architecture of FTO/IJP-SnO_*x*_/IJP-MHP/IJP-spiro-OMeTAD/Au. This best performance was obtained using perovskite films printed from the DMF/DMSO/ACN solvent system. This efficiency was comparable to and even higher than those for n-i-p PSCs with inkjet-printed perovskite layers and inkjet-printed charge transport layers reported previously. Compared to the spin-coated PSCs, the inferior performance of the inkjet-printed devices was due to poor surface morphology and inhomogeneity of the film thickness, which originated from the rapid nucleation and grain growth of the perovskite films during inkjet printing. The PVP additive and antisolvent bath treatment were then carried out to retard perovskite nucleation and grain growth. Smoother perovskite films with improved film morphology and thickness homogeneity were obtained in either the DMF/DMSO-PVP-Anti or DMF/NMP-PVP-Anti case, although these perovskite films exhibited low PCEs due to the insulating properties of PVP. Further investigations can be conducted to boost PCEs to higher levels by replacing PVP with a conductive additive or that can be removed by the following antisolvent bath treatment. This work paves the way for scalable fabrication of PSCs with inkjet-printed perovskite layers and inkjet-printed charge transport layers.

## 4 Experimental Methods

### 4.1 Materials

All chemicals were used as received, with no modifications. Tin(IV) acetate (Sn(CH_3_CO_2_)_4_) for making ETL precursor inks was purchased from Sigma–Aldrich (Darmstadt, Germany). Lead iodide (PbI_2_, 99.99%) and lead bromide (PbBr_2_, >98.0%) were purchased from TCI (Tokyo, Japan). Formamidinium iodide (FAI, CH(NH_2_)_2_I, >98%) and methylammonium bromide (MABr, CH_3_NH_3_Br, >98%) were purchased from Dyenamo (Stockholm, Sweden) and Sigma–Aldrich (Darmstadt, Germany), respectively. Spiro-OMeTAD (99.8%) was purchased from Borun New Material Technology (Ningbo, China). Bis(trifluoromethane)sulfonimide lithium salt (LiTFSI, 99.95%), FK209 (Co (III) TFSI salt, 98%), and 4-*tert*-butylpyridine (TBP, 98%) were purchased from Sigma–Aldrich (Darmstadt, Germany). Solvents for perovskite precursor inks were obtained from Sigma–Aldrich (Darmstadt, Germany), such as *N*,*N*-dimethylformamide (DMF, anhydrous, 99.8%), dimethyl sulfoxide (DMSO, anhydrous, ≥99.9%), acetonitrile (ACN, anhydrous, 99.8%), 2-methoxyethanol (MOE, ≥99.9%), and *N*-methyl-2-pyrrolidinone (NMP, anhydrous, 99.5%). Toluene (anhydrous, 99.8%) for spiro-OMeTAD precursor inks, perovskite precursor additive polyvinylpyrrolidone (PVP, molar weight 40,000), and antisolvent chlorobenzene (anhydrous, 99.8%) were also purchased from Sigma–Aldrich (Darmstadt, Germany).

### 4.2 Set-Up of Inkjet Printer

A drop-on-demand inkjet printing system was designed and used under ambient conditions in our laboratory.^50^ Inkjet printing of perovskite films and charge transport layers was performed using XJ126/80 printheads (XAAR) with 126 active nozzles and a drop volume of 80 pL. A customized waveform was used to drive the printheads. The printing frequency was set to 283.46 Hz, and the printing resolution was 360 dpi.

### 4.3 VA and Self-VA Treatment

The thermal annealing temperature was fixed at 100 °C, and the total annealing time was 30 min for each sample. For VA treatment, 1 μL of DMSO was dripped onto the hot plate and immediately covered with a glass Petri dish. An as-prepared perovskite was inserted into the Petri dish, which was filled with the DMSO solvent vapor. VA treatment was carried out in the Petri dish for 25 min, and then, the perovskite film was thermally annealed for another 5 min after the Petri dish was removed. A self-VA treatment was carried out in a Petri dish without the addition of any additional solvent. The perovskite film was exposed to the solvent vapor evaporated from itself. This self-VA treatment was also carried out in the Petri dish for 25 min, and the perovskite film was thermally annealed for an additional 5 min after removal of the Petri dish.

### 4.4 Inkjet Printing of Perovskite Layers

For in situ heat treatment, the perovskite precursor was prepared by dissolving PbI_2_, FAI, PbBr_2_, and MABr in a mixed solvent (DMF/DMSO = 4/1, v/v) to form a 0.65 M precursor ink with the four components in a molar ratio of 1.1:1:0.2:0.2. Before inkjet printing, the substrates were heated to various temperatures of 60, 80, 100, 120, and 150 °C. After finishing printing, the resulting perovskite films were immediately transferred to a hot plate and dried at 100 °C for 30 min. For perovskite films with self-VA treatment, the as-deposited perovskite film was annealed inside a glass Petri dish at 100 °C for 25 min and then thermally annealed for another 5 min after the Petri dish.

For perovskite films prepared with DMF/DMSO/ACN or DMF/DMSO/MOE solvent systems, PbI_2_, FAI, PbBr_2_, and MABr were dissolved in a mixed solvent of DMF and DMSO with a volume ratio of 4:1. The molar ratio of PbI_2_:FAI:PbBr_2_:MABr was fixed at 1.1:1:0.2:0.2. Before printing, ACN or MOE was added to the perovskite solution in a volume fraction of 20% of the final precursor ink, which was then stirred magnetically for 1 h. Then, the perovskite ink was inkjet-printed at a substrate temperature of 150 °C, and the as-deposited perovskite film was annealed at 100 °C with self-VA treatment.

For the perovskite film with the PVP additive and antisolvent treatment, the four perovskite components were dissolved in a mixed solvent of DMF/DMF or DMF/NMP (4/1, v/v) in a molar ratio of 1.1:1:0.2:0.2. PVP was added in a weight fraction of 5% of PbI_2_. The perovskite ink was printed at a substrate temperature of 80 °C. The as-deposited wet perovskite film was immersed into an antisolvent (chlorobenzene) bath for 2 min and, afterward, was annealed at 100 °C for 30 min.

### 4.5 Device Fabrication

Fluorine-doped tin oxide (FTO) glass substrates (14 Ω/sq, Pilkington TEC) were cut into pieces with a size of 25 × 15 mm. Each piece was etched at the edge with Zn powder and a 2 M HCl aqueous solution. These substrates were successively sonicated in a detergent solution (5% deconex in water), deionized water, acetone, and 2-propanol for 15 min. Before the inkjet printing of ETL, FTO substrates were preheated at 500 °C for 30 min and afterward cooled down to room temperature. SnO_*x*_ ETLs were fabricated following the procedure reported in our previous work.^40^ SnO_*x*_ thin films were inkjet-printed from a 0.05 M ink with tin(IV) acetate dissolved in a mixture of 2-propanol and propylene glycol (9/1, v/v). A small amount of ethanolamine was added to improve the solubility of tin acetate. For printing, a substrate was placed on a preheated printing stage at 60 °C; 5 min after printing, the substrate was transferred to a furnace and annealed at 220 °C for 1 h. The perovskite absorber layers were fabricated as described above. The as-deposited perovskite films were dried at 100 °C for 25 min inside a Petri dish and thermally annealed for another 5 min after the cover. A hole transport layer was inkjet-printed with a precursor consisting of 17.5 mM spiro-OMeTAD, 5 mM LiTFSI, 50 mM TBP, and 0.5 mM FK 209 in toluene. The substrate temperature was set as 60 °C, and then, the resultant spiro-OMeTAD film was obtained without further thermal annealing. Finally, a Au electrode with a thickness of 80 nm was deposited via thermal evaporation (Edwards Auto 306).

### 4.6 Characterization

XRD data were obtained by using an X-ray diffractometer (Siemens D5000, Siemens, Munich, Germany) with Cu Kα1 radiation (λ = 1.5406 Å). The film morphology and cross-sectional structures were examined by using a combined focused ion beam/scanning electron microscope (FIB/SEM, FEI Nova 600 Nanolab, FEI Company, Eindhoven, The Netherlands). The working area of the solar cells was defined by a mask of 0.126 cm^2^ and illuminated under an AM 1.5G solar simulator (Newport 91160-1000), with an incident light density of 100 mW/cm^2^. Photocurrent density–voltage (*J*–*V*) characteristics were collected by using a Keithley 2400 unit with a scan rate of 125 mV/s. Time-resolved photoluminescence (TRPL) of perovskite films was investigated using a home-built system, as described previously.^51,52^ When correcting for laser scatter and removing contributions from the laser pulse, time constants as short as 4 ns could be accurately determined. A pulsed nanosecond laser with a wavelength of 450 nm (Thorlabs, NPL45C) excited the sample, and detection was made using a photomultiplier tube (Thorlabs, PMTSS). The TRPL decay curve can be fitted with a biexponential decay function:
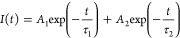
1where τ_1_ and τ_2_ represent the fast and slow decay components, and *A*_1_ and *A*_2_ are the corresponding decay amplitude fractions, respectively. The average lifetime (τ_ave_) was determined by the following formula:
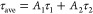
2The fast decay component, τ_1_, can be attributed to the trapping of photogenerated charge carriers at the perovskite-glass interface, where surface states or interfacial defects act as quenching sites. The slow decay component, τ_2_, primarily corresponds to radiative recombination of free charge carriers in the perovskite film, though a minor contribution from nonradiative recombination processes, such as Shockley–Read–Hall recombination, cannot be excluded.

## Supporting Information Available

Diagram of the inkjet printing process, XRD patterns, distributions of photovoltaic parameters, SEM images, TRPL decay traces, detailed photovoltaic parameters, FWMH and peak intensity ratios extracted from XRD, and TRPL decay lifetimes (DOCX)
