# Supplementary material for: Scalable Fabrication of Perovskite Solar Cells with Inkjet-Printed Perovskite Absorbers Processed under Ambient Conditions
Source: ACS Appl Mater Interfaces. 2025 Apr 29;17(19):28055–64. doi: 10.1021/acsami.4c20567 (PMC12086759; doi:10.1021/acsami.4c20567)
Supplement: Supplementary file 1 — am4c20567_si_001.docx [file am4c20567_si_001.docx]

Supporting Information

Scalable Fabrication of Perovskite Solar Cells with Inkjet-Printed Perovskite Absorbers Processed under Ambient Conditions

Dongli Lu,*^,†^ Mahboubeh Jamshidi,^‡^ James M. Gardner,^‡^ and Liubov Belova*^,†^

^†^Department of Materials Science and Engineering, KTH Royal Institute of Technology, Stockholm 10044, Sweden

^‡^Department of Chemistry, Applied Physical Chemistry, KTH Royal Institute of Technology, Stockholm 10044, Sweden

*E-mail: donglil@kth.se (D. Lu), lyuba@kth.se (L. Belova)


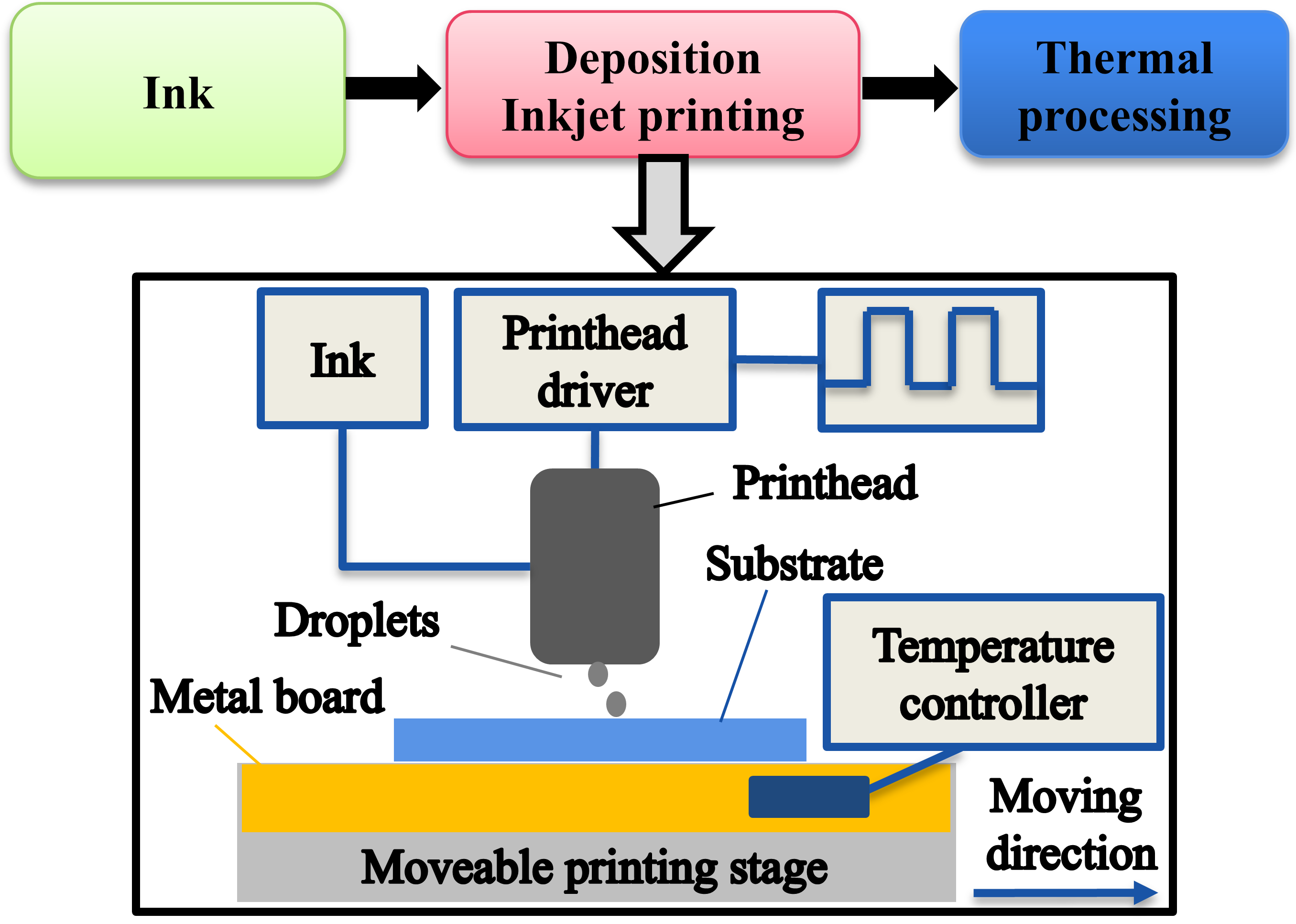


**Figure S1.** Diagram of the inkjet printing of perovskite films.


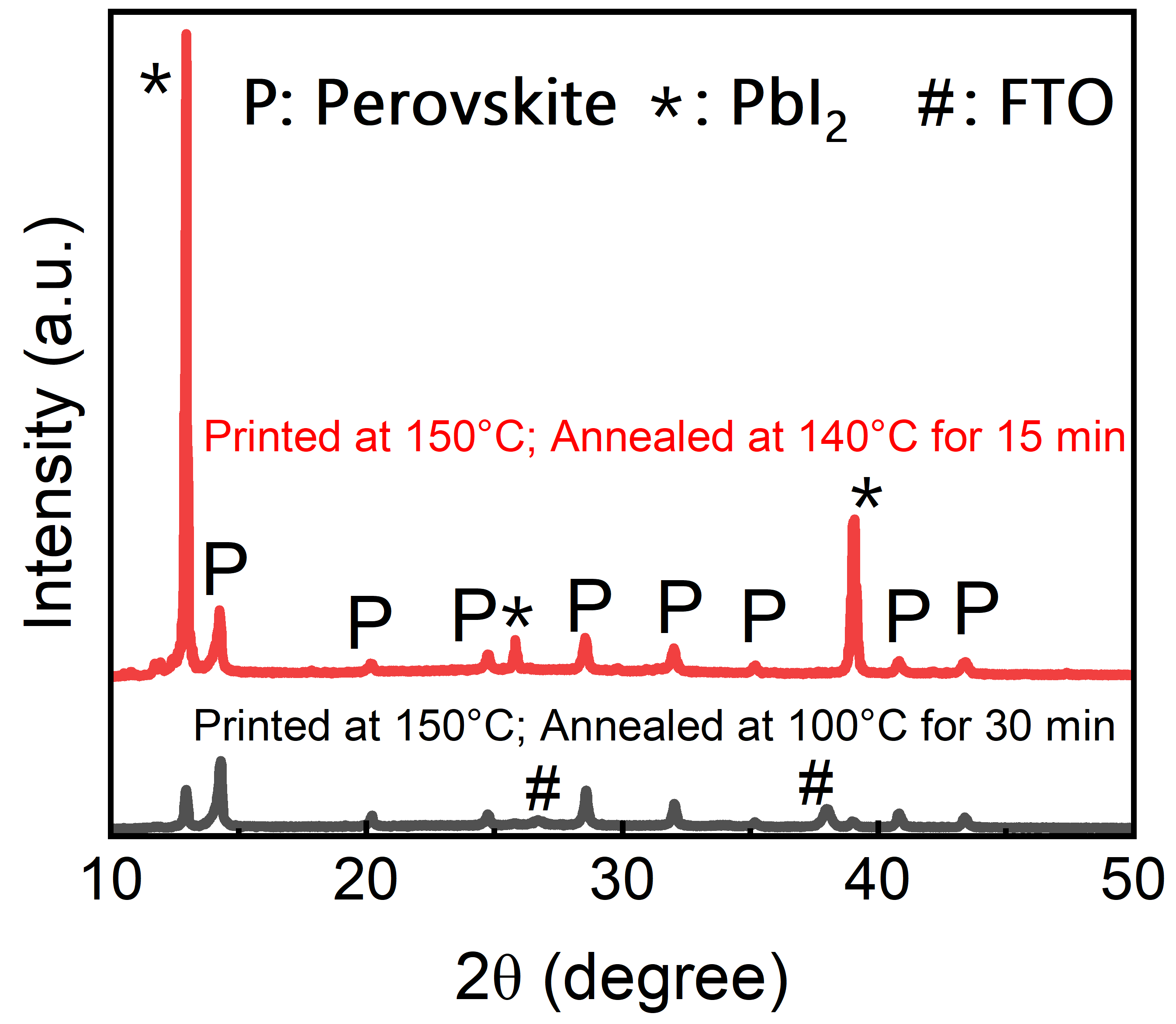


**Figure S2.** XRD patterns of inkjet-printed perovskite films annealed at different temperatures.


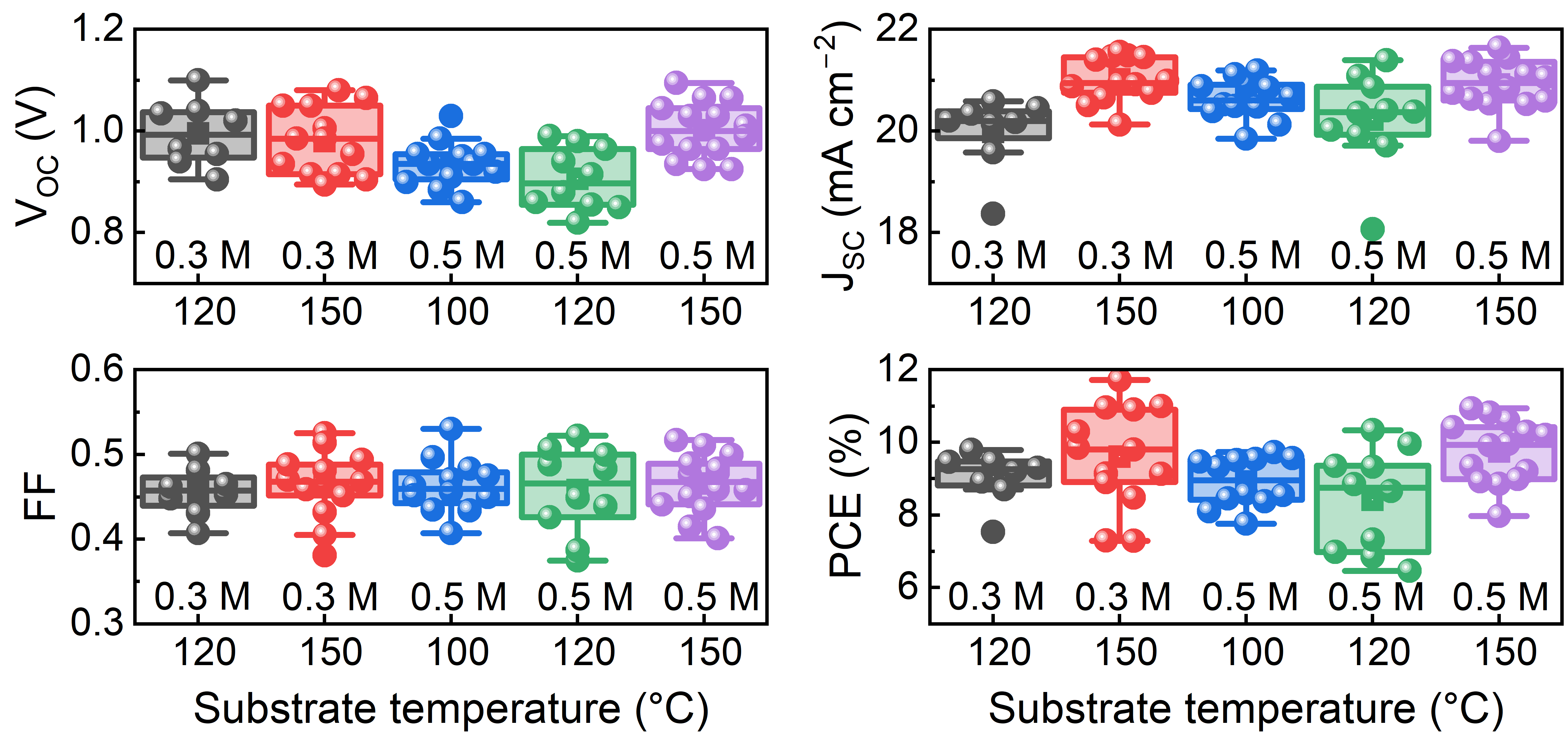


**Figure S3.** Distributions of photovoltaic parameters for the cells with perovskite films printed at different substrate temperatures.


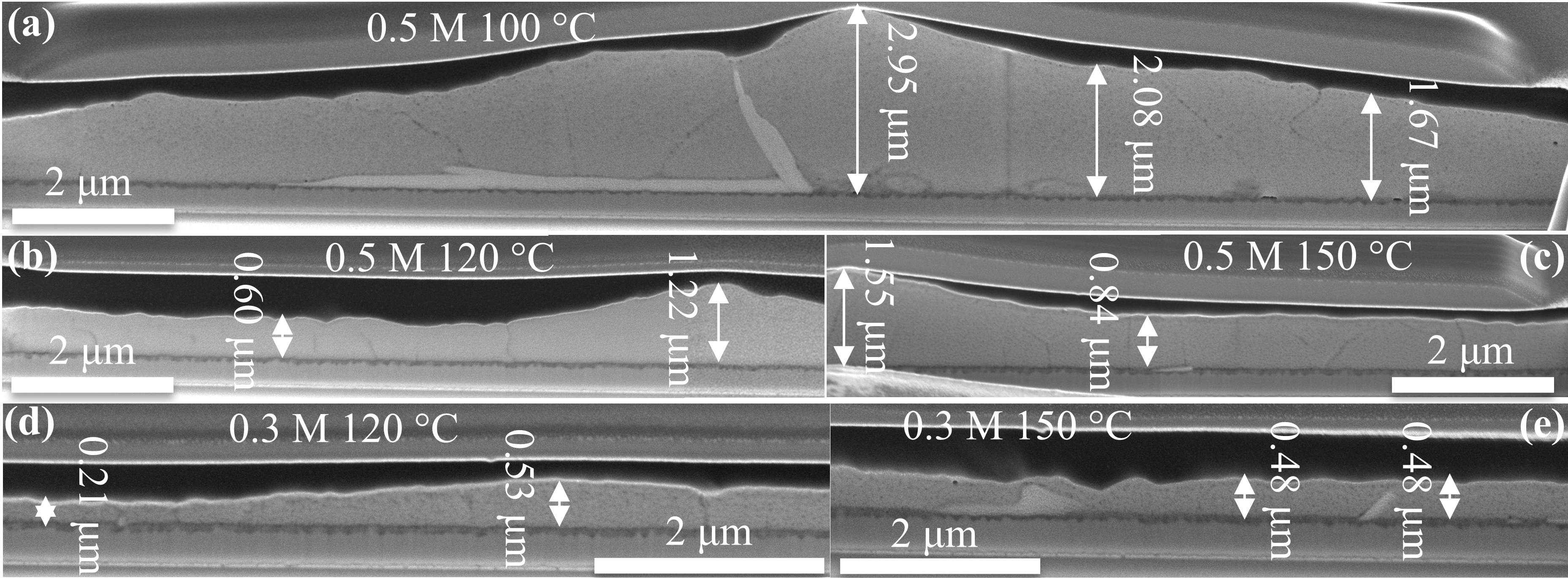


**Figure S4.** Cross-sectional FIB/SEM images of PSCs with perovskite films printed at different substrate temperatures (a) 100 °C, (b) 120 °C, and (c) 150 °C for 0.5 M inks, and (d) 120 °C and (e) 150 °C for 0.3 M inks.


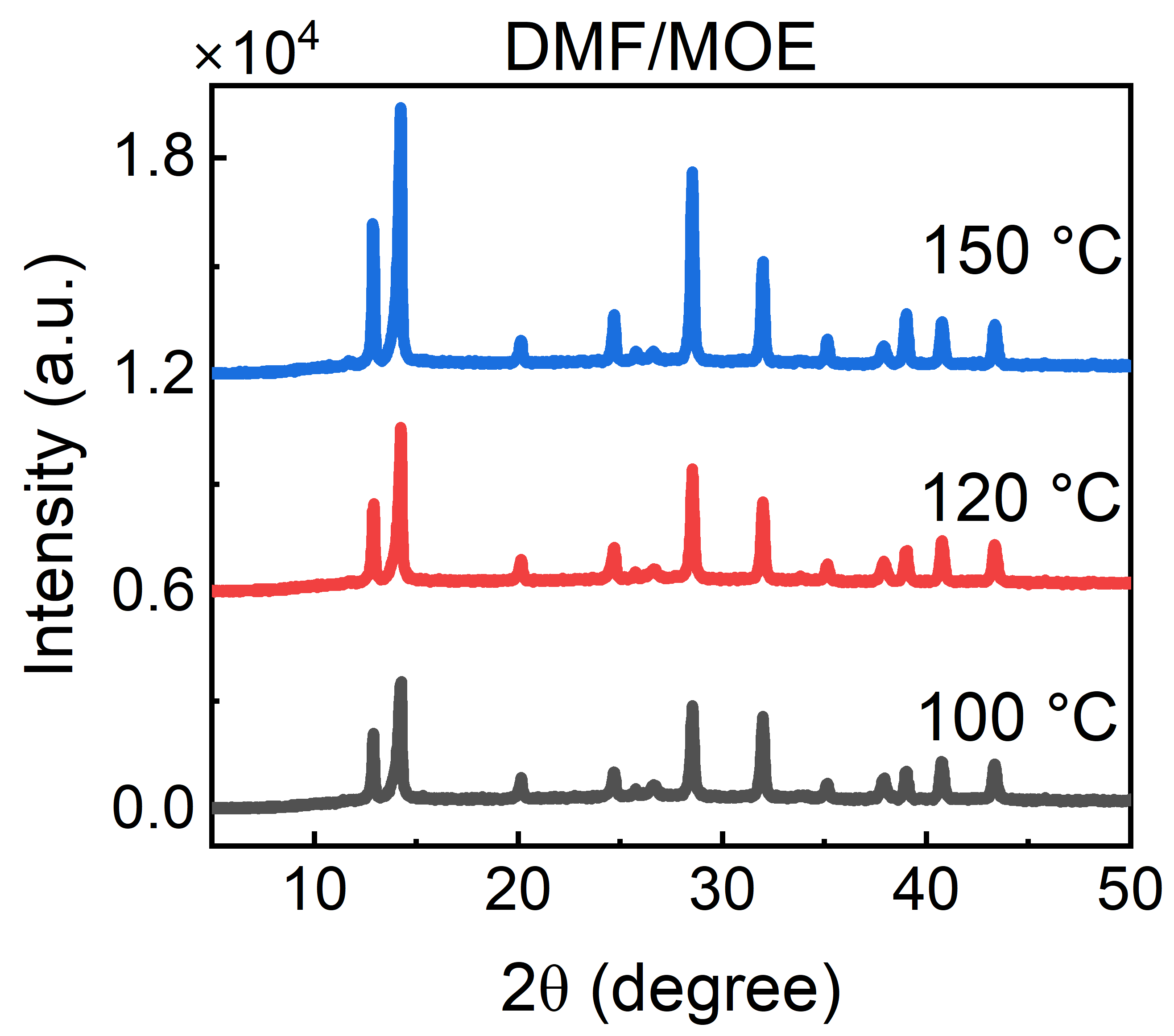


**Figure S5.** XRD patterns of perovskite films with the DMF/MOE solvent system.


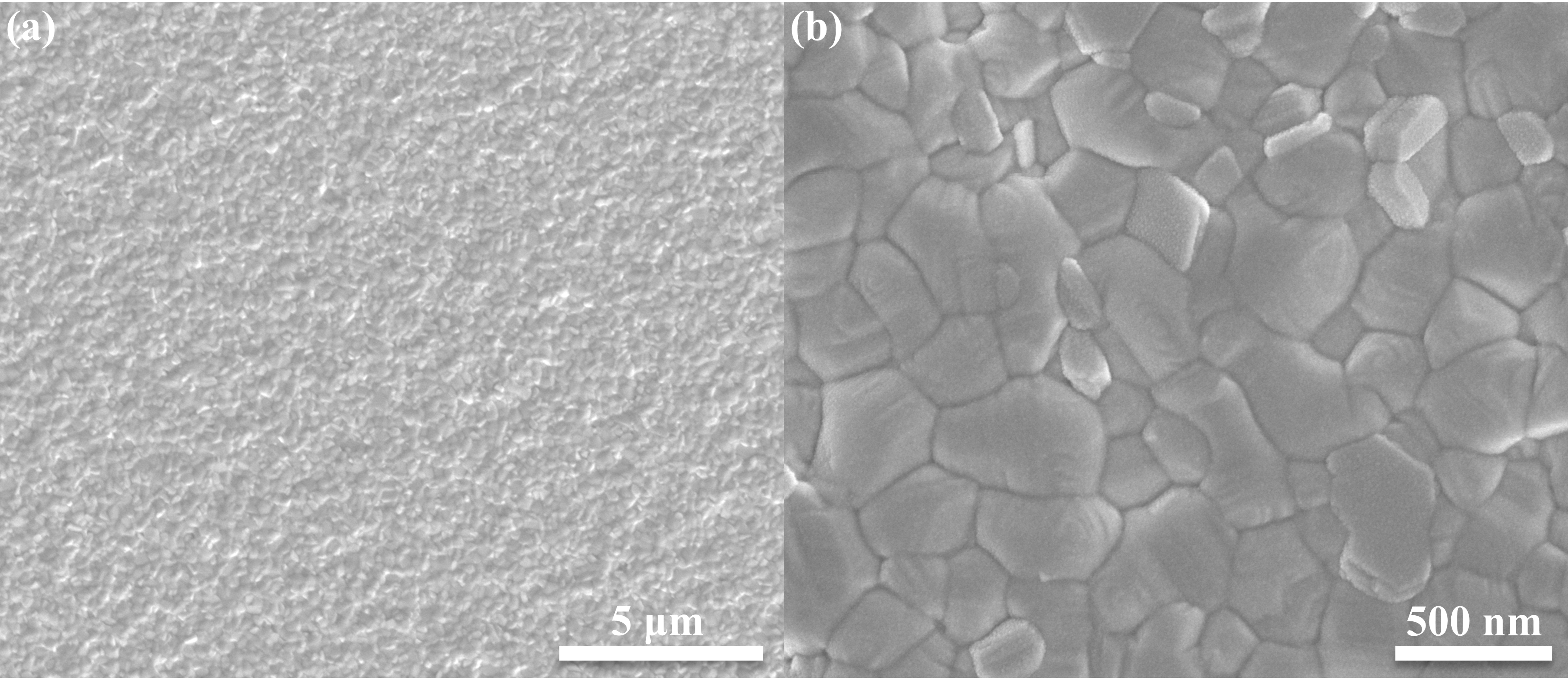


**Figure S6.** (a) Top-view SEM image of the spin-coated perovskite layer and (b) an expanded image at higher magnification.


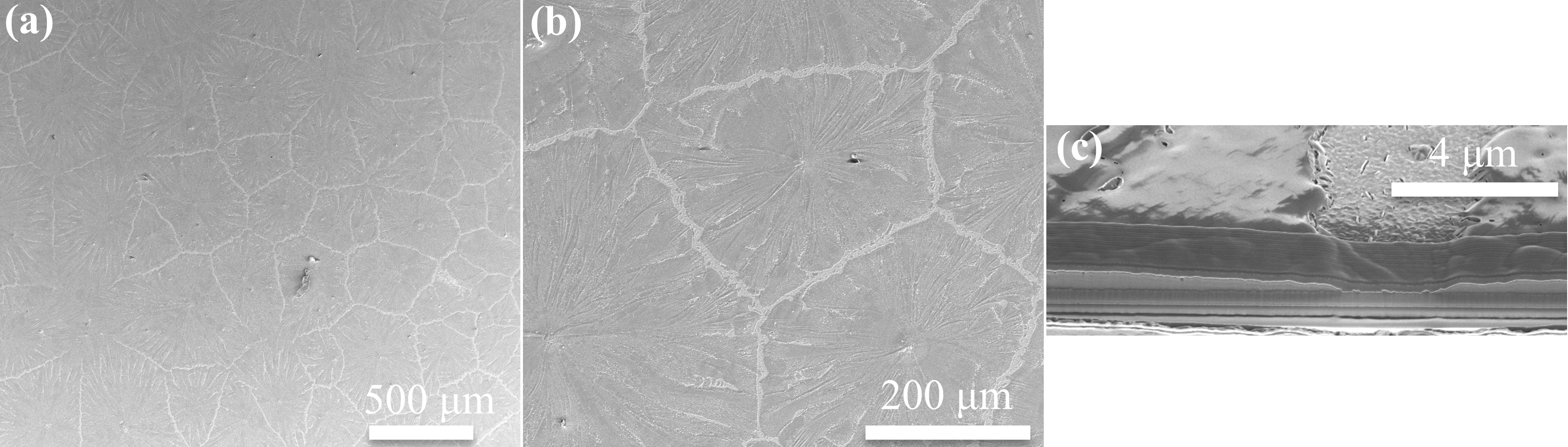


**Figure S7.** (a-b) Top-view and (c) cross-sectional SEM images of the inkjet-printed perovskite layer on top of the SnO_x_ layer when the substate temperature was 80 °C.


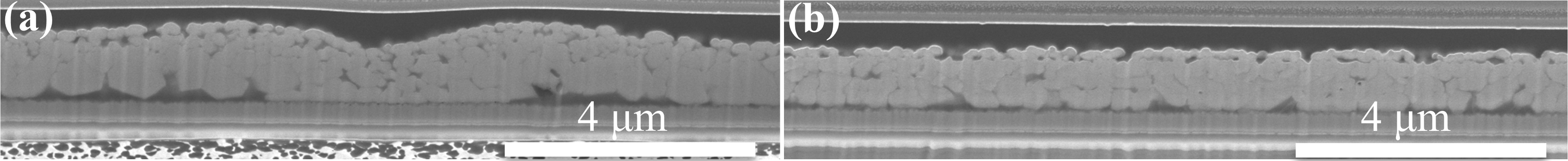


**Figure S8.** Cross-sectional SEM images of perovskite films with the DMF/DMSO-PVP solvent system and antisolvent treatment (a), and with the DMF/NMP-PVP solvent system and antisolvent treatment (b).


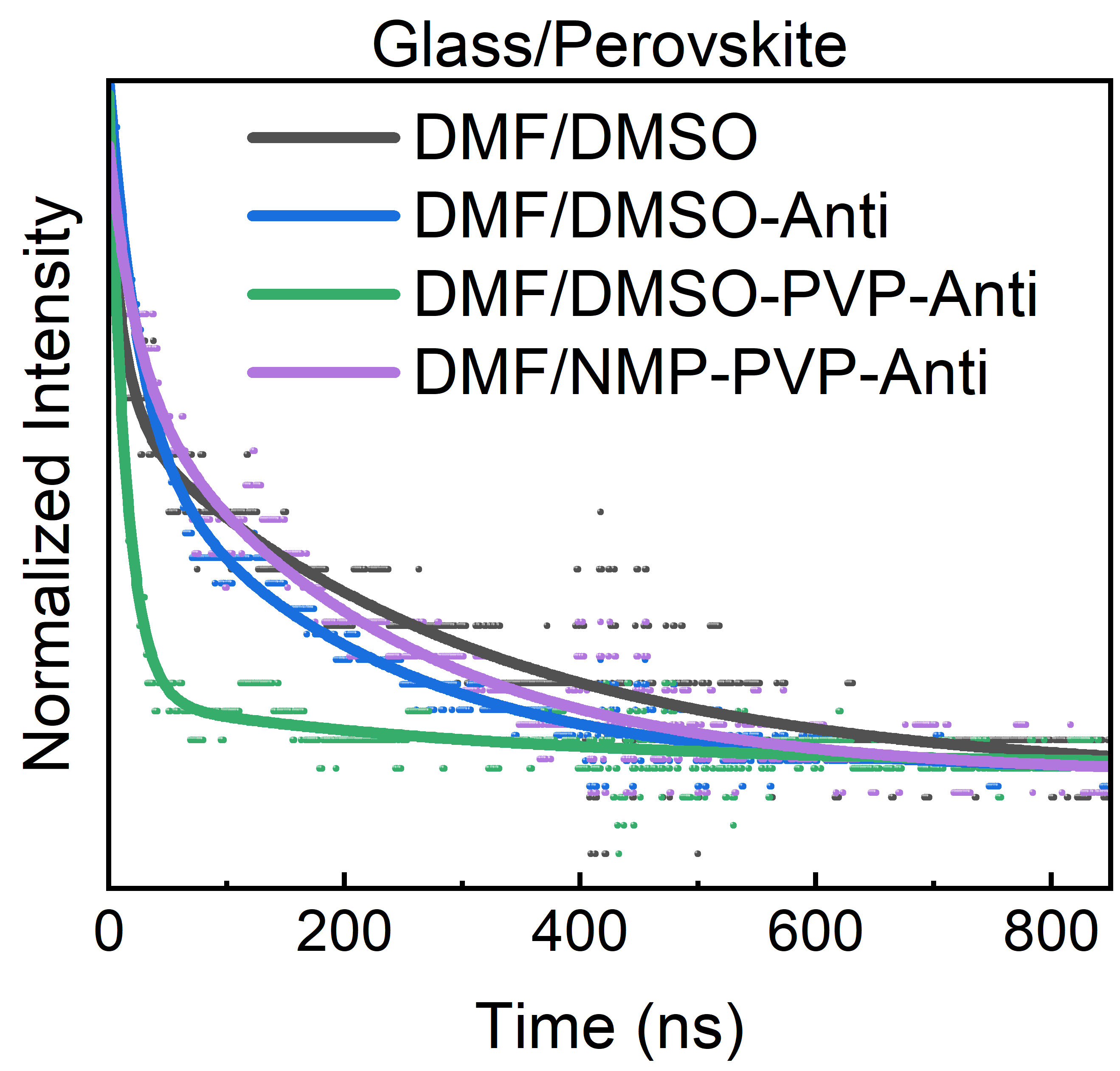


**Figure S9.** TRPL decay traces of inkjet-printed perovskite films prepared with the additive PVP and antisolvent treatment.

**Table S1.** Photovoltaic parameters of the cells using inkjet-printed perovskite films with the in situ heat treatment at different substrate temperatures.

| Samples | Scan direction | PCE (%) | V_OC_ (V) | J_SC_ (mA/cm^2^) | FF |
| --- | --- | --- | --- | --- | --- |
| 0.3 M 120 °C | reverse  (average) | 9.80  (9.05 ± 0.70) | 1.04  (1.00 ± 0.06) | 20.34  (19.98 ± 0.71) | 0.465  (0.456 ± 0.029) |
|  | forward | 8.03 | 1.01 | 20.57 | 0.386 |
| 0.3 M 150 °C | reverse  (average) | 11.71  (9.60 ± 1.39) | 1.08  (0.98 ± 0.07) | 20.66  (21.01 ± 0.44) | 0.525  (0.464 ± 0.040) |
|  | forward | 9.35 | 1.03 | 21.16 | 0.429 |
| 0.5 M 100 °C | reverse  (average) | 9.73  (8.92 ± 0.68) | 0.96  (0.94 ± 0.05) | 20.49  (20.63 ± 0.40) | 0.497  (0.463 ± 0.032) |
|  | forward | 7.76 | 0.90 | 20.55 | 0.422 |
| 0.5 M 120 °C | reverse  (average) | 10.34  (8.41 ± 1.39) | 0.99  (0.91 ± 0.06) | 20.87  (20.22 ± 0.92) | 0.500  (0.458 ± 0.051) |
|  | forward | 8.84 | 0.93 | 20.85 | 0.456 |
| 0.5 M 150 °C | reverse  (average) | 10.94  (9.75 ± 0.88) | 1.03  (1.00 ± 0.06) | 20.53  (20.92 ± 0.48) | 0.517  (0.465 ± 0.035) |
|  | forward | 9.33 | 0.99 | 20.94 | 0.452 |

**Table S2.** The full width at half maximum height (FWMH) at the characteristic peak 14.3° and the intensity ratio of 14.3°/20.2° extracted from XRD patterns of perovskite films printed from the DMF/DMSO and DMF/DMSO/ACN solvent system, respectively.

|  |  | 100 °C | 120 °C | 150 °C |
| --- | --- | --- | --- | --- |
| DMF/DMSO | FWMH (rad) | 0.0041 | 0.0043 | 0.0040 |
|  | Intensity ratio of 14.3°/20.2° | 7.1 | 7.0 | 5.1 |
| DMF/DMSO/ACN | FWMH (rad) | 0.0042 | 0.0046 | 0.0045 |
|  | Intensity ratio of 14.3°/20.2° | 7.1 | 7.2 | 5.0 |

**Table S3.** Photovoltaic parameters of the PSCs with spin-coated perovskite layers and with inkjet-printed perovskite layers prepared from two different solvent systems DMF/DMSO/ACN and DMF/DMSO/MOE.

| Sample | Scan direction | PCE (%) | V_OC_ (V) | J_SC_ (mA/cm^2^) | FF | HI (%) |
| --- | --- | --- | --- | --- | --- | --- |
| Spin-coated | reverse | 14.97  (13.51 ± 0.79) | 1.09  (1.05 ± 0.02) | 20.64  (20.63 ± 0.34) | 0.669  (0.626 ± 0.030) | 22.6  (17.7 ± 2.3) |
|  | forward | 11.59  (11.11 ± 0.58) | 1.03  (0.99 ± 0.02) | 20.91  (20.65 ± 0.28) | 0.538  (0.542 ± 0.024) |  |
| DMF/DMSO/ACN | reverse | 13.44  (11.45 ± 1.02) | 1.10  (1.04 ± 0.05) | 21.50  (21.08 ± 0.47) | 0.568  (0.522 ± 0.031) | 25.6  (24.3 ± 3.3) |
|  | forward | 10.00  (8.66 ± 0.74) | 1.06  (0.97 ± 0.05) | 21.40  (21.04 ± 0.49) | 0.443  (0.423 ± 0.029) |  |
| DMF/DMSO/MOE | reverse | 11.82  (10.74 ± 0.94) | 1.03  (1.01 ± 0.05) | 21.72  (20.98 ± 0.88) | 0.531  (0.509 ± 0.036) | 16.3  (19.3 ± 2.9) |
|  | forward | 9.90  (8.66 ± 0.67) | 0.97  (0.95 ± 0.06) | 21.89  (21.07 ± 0.81) | 0.468  (0.436 ± 0.034) |  |

**Table S4.** TRPL decay lifetimes of inkjet-printed perovskite films prepared with the DMF/DMSO solvent system, with the DMF/DMSO solvent system and antisolvent treatment, with the DMF/DMSO-PVP solvent system and antisolvent treatment, and with the DMF/NMP-PVP solvent system and antisolvent treatment.

|  |  | A_1_ | τ_1_ (ns) | A_2_ | τ_2_ (ns) | τ_ave_ (ns) |
| --- | --- | --- | --- | --- | --- | --- |
|  | DMF/DMSO | 0.38 | 14.3 | 0.62 | 297.0 | 189.7 |
| Glass/Perovskite | DMF/DMSO-Anti | 0.51 | 25.0 | 0.49 | 204.0 | 112.7 |
|  | DMF/DMSO-PVP-Anti | 0.91 | 14.6 | 0.09 | 248 | 35.6 |
|  | DMF/NMP-PVP-Anti | 0.36 | 23.1 | 0.64 | 220.7 | 149.6 |

**Table S5.** Photovoltaic parameters of the cells using perovskite films prepared with the DMF/DMSO solvent system, with the DMF/DMSO solvent system and antisolvent treatment, with the DMF/DMSO-PVP solvent system and antisolvent treatment, and with the DMF/NMP-PVP solvent system and antisolvent treatment.

| Sample | PCE (%) | V_OC_ (V) | J_SC_ (mA/cm^2^) | FF |
| --- | --- | --- | --- | --- |
| DMF/DMSO | 9.68  (8.60 ± 0.94) | 0.91  (0.88 ± 0.01) | 19.77  (18.86 ± 1.11) | 0.538  (0.516 ± 0.02) |
| DMF/DMSO-Anti | 0.74  (0.59 ± 0.13) | 0.52  (0.52 ± 0.04) | 5.47  (4.26 ± 0.83) | 0.263  (0.265 ± 0.017) |
| DMF/DMSO-PVP-Anti | 2.51  (1.22 ± 0.70) | 0.95  (0.91 ± 0.03) | 7.22  (4.06 ± 2.05) | 0.367  (0.320 ± 0.020) |
| DMF/NMP-PVP-Anti | 0.38  (0.25 ± 0.10) | 0.82  (0.77 ± 0.05) | 2.07  (1.38 ± 0.50) | 0.227  (0.232 ± 0.023) |
